# Longitudinal Changes in the Brain Following Third Ventriculostomy in a Child With Hydrocephalus: A Case Report

**DOI:** 10.1097/MD.0000000000002095

**Published:** 2015-10-30

**Authors:** Yongxin Li, Cailei Zhao, Zhen Tan, Ya Wang, Heye Zhang, Jinyang Wang, Honghua Guo, Baozhen Zeng, Wenhua Huang

**Affiliations:** From the Institute of Clinical Anatomy, Southern Medical University, Guangzhou (YL, YW, JW, HG, BZ, WH); Department of Pediatric Radiology, Shenzhen Children Hospital (CZ); Department of Pediatric Neurosurgery, Shenzhen Children Hospital (ZT); and Shenzhen Institutes of Advanced Technology, Chinese Academy of Sciences, Shenzhen, China (HZ).

## Abstract

The goal of this study was to detect the long-term effect of shunting on the integrity of white matter in young children with hydrocephalus.

The authors reported the case of a 6-month-old boy with hydrocephalus who was evaluated by diffusion tensor imaging (DTI) before and after a shunt operation.

When compared with normal children, the structures of the corpus callosum, internal capsule, and corona radiata in the patient showed a decrease in fractional anisotropy and an increase in radial diffusivity values before the shunt operation. Following successful cerebrospinal fluid shunting, long-term follow-up DTI demonstrated a trend toward normalization of the fractional anisotropy and radial diffusivity values.

Shunt treatment can prevent further damage to the brain and grossly reconstitute the distorted anatomy. DTI could be a useful tool in detecting longitudinal changes after a shunt operation. Further studies involving larger case numbers are needed to detect the long-term effect of shunting on the brains of children with hydrocephalus.

## INTRODUCTION

Hydrocephalus is a severe pathologic condition that is characterized by an imbalance in the production and absorption of cerebrospinal fluid (CSF). It is accompanied by expansion of the ventricles and displacement of adjacent brain structures. Previous studies of hydrocephalus have found that the structural and functional alterations appear to occur due to the mechanical distortion of the brain.^1,2^ Behaviorally, a variety of cognitive deficits are present in these hydrocephalus patients, including reductions in intelligence, motor skills, memory, visuospatial skills, and language.^1^

Currently, the CSF shunt is the only effective treatment for most patients with hydrocephalus. The treatment is undertaken with the assumption that draining the CSF can prevent further damage to the brain, thereby reversing part of the injury and improving brain function.^3–5^ Previous studies have shown that shunt surgery can grossly reconstitute distorted anatomy and improve cognitive function.^2,5–8^ Although the shunting effect on the brain has been studied, most of these studies have focused on a short follow-up period time, such as several weeks or months. Little is known about the long-term effects of hydrocephalus on the brain after shunt treatment.

In the present study, we aimed to extend these previous findings and explore white matter (WM) longitudinal alterations in hydrocephalus. A pediatric patient with hydrocephalus was evaluated by diffusion tensor imaging (DTI) both before and after a shunt operation. DTI is an advanced neuroimaging technique that can measure in vivo WM structural integrity.^9^ For this reason, we used this technique to evaluate the longitudinal changes of WM in hydrocephalus with shunt treatment. Fractional anisotropy (FA) and radial diffusivity (RD) were used to quantify the microscopic diffusion properties of WM. A region-of-interest (ROI) analysis was used to investigate the neurodevelopmental trajectory of WM following shunting in a patient with hydrocephalus.

## CASE REPORT

### Subjects

One patient with hydrocephalus and 10 right-handed, age-matched control subjects (10 males; age range 4–44 months) participated in this study. None of the controls had a history of neurologic or psychiatric disorders. The patient was a 6-month-old, right-handed, male child. This patient was diagnosed with noncommunicating hydrocephalus through neuroimaging technology. The fourth ventricle outlet obstruction was detected using magnetic resonance imaging (MRI) and computed tomography ventricular angiography. His parents reported a history of vomiting and reduced appetite to the Children's Hospital of Shenzhen. The signs and symptoms of this patient included an enlarged head circumference, anterior fontanelle uplift, and high tension, and his motor development was reduced. He was scanned using a T2-weighted imager to design surgical planning. The patient underwent a third ventriculostomy for treatment of hydrocephalus. Following the operation, he was regularly examined. The symptoms of vomiting and reduced appetite disappeared. Although the head circumference of the patient remained enlarged, the anterior fontanelle became flattened with reduced tension. Two years postsurgery, the patient can walk and run. In addition, he can speak using simple words, such as goodbye and hello.

The Ethics Committee of the Children's Hospital of Shenzhen approved the study protocol. The families of the participants provided written informed consent when enrolled into the study.

### Image Acquisition

**FIGURE 1:**
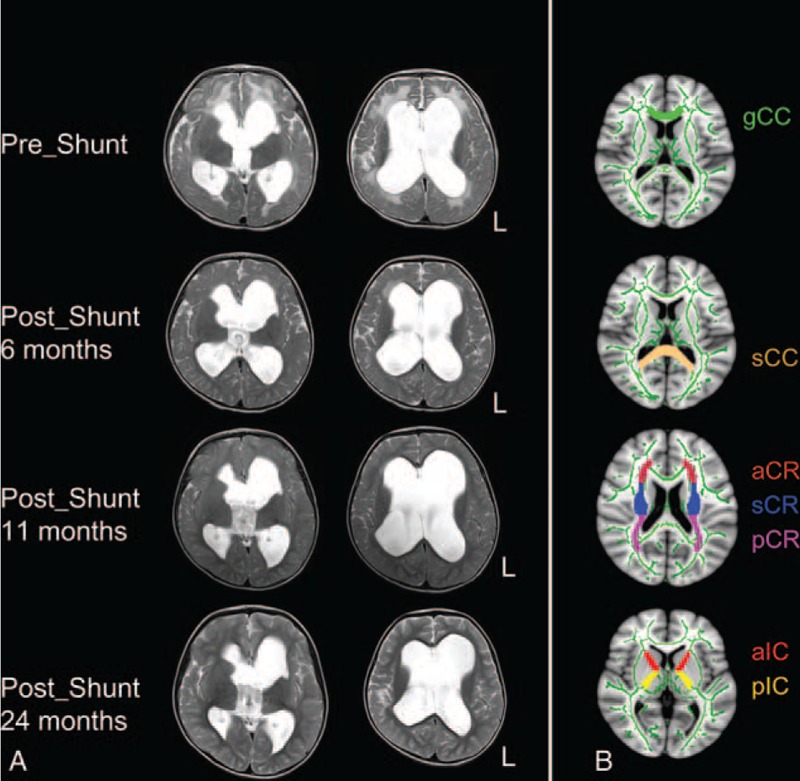
Patient's T2-weighted images and the location of the ROIs.

DTI data were collected using a 3T Siemens scanner (MAGNETOM Trio Tim; Siemens, Germany) at the Children's Hospital of Shenzhen, Shenzhen, China. T2-weighted images of the patient were collected to diagnose the brain abnormality (Figure 1A). DTI was performed 4 times in the patients (the first DTI before the shunt, the second DTI at 6 months after the shunt, the third DTI at 11 months after the shunt, and the fourth DTI at 24 months after the shunt) and once in the control subjects. The DTI acquisition used a spin-echo planar image sequence with the following parameters: repetition time = 6.8 s, echo time = 93 ms, NEX = 3, flip angle = 90°, field of view = 220 × 220 mm^2^, in plane resolution = 1.719 × 1.719 mm^2^, 40 axial slices, slice thickness = 2.5 mm. Diffusion-sensitized images were collected in 30 diffusion directions with b = 1000 s/mm^2^ along with 1 nondiffusion-weighted volume. Foam cushions were used to reduce head translation movement and rotation.

The phase-contrast (PC) MRI data for this patient were also collected to see the CSF dynamics at different scanning times. The CSF flow was measured to evaluate the shunting result.

### Imaging Processing and Statistical Analysis

Image processing and analysis were performed by using the FMRIB Software Library (University of Oxford, FSL v5.0.1, www.fmrib.ox.ac.uk/fsl). Standard processing steps were used.^10^ First, images were corrected for eddy currents and head motion correction using affine registration.^11^ FMRIB's Brain Extraction Tool (BET v2.1) was then used for brain extraction.^12^ Subsequently, eigenvectors and eigenvalues (λ1, λ2, and λ3) of diffusion tensors were estimated at each voxel.^13^ FA and RD (corresponds to (λ2 + λ3)/2) were calculated using FMRIB's Diffusion Toolbox (FDT v3.0) to characterize microstructural features of WM.

Certain scripts in TBSS (a part of FSL^14^) were used to convert the registration and transformation into the standard space. All subjects’ FA images were first aligned to each other to identify the “most representative” one as the target image. This target image was then affine-aligned into MNI152 standard space. Each subject's FA image was nonlinearly transformed into the target image, and then the affine transform to 1 x 1 x 1 mm MNI152 space was applied, resulting in a transformation of the original FA image into MNI152 space. The same processing protocol was conducted with the RD image.

As seen in Figure 1B, the regions-of-interest (ROIs) included the genu of the corpus callosum (gCC), the splenium of the corpus callosum (sCC), the anterior limb of the internal capsule (aIC), the posterior limb of the internal capsule (pIC), the anterior corona radiata (aCR), the superior corona radiata (sCR), and the posterior corona radiata (pCR). These regions were defined using the Johns Hopkins University ICBM-DTI-81 WM atlas.^15^ The mean FA and RD values in each ROI were extracted from both the patient and normal control subjects.

## RESULTS

DTI results are summarized in Table 1. In the gCC and sCC, the FA values of the pre-shunt DTI were decreased by 29% and 40%, respectively, compared with the mean FA values of the normal controls. Six months after the shunt operation, the FA values were increased by 12% and 15%; however, the FA values at 11 months postshunt were decreased to the pre-shunt level. At 24 months postshunt, the FA values were increased but still far from the normal control values (equal or over 2 standard deviations). The RD values in the gCC and sCC before the shunt were increased by 53% and 34%, compared with the controls. After the shunt, the RD values in these ROIs decreased with time. At 24 months postshunt, the RD values were decreased and close to those of the normal controls (less than 2 standard deviations).

**TABLE 1:**
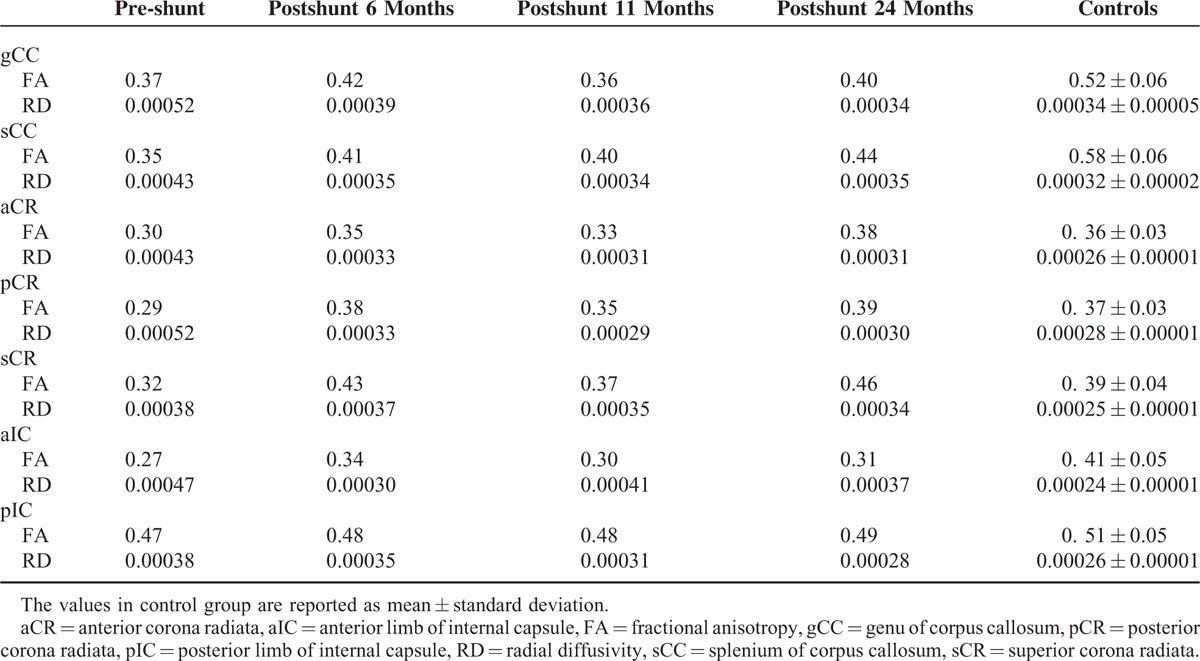
Comparison of DTI Parameters of the Patients and the Controls in ROIs

In the ROIs of the corona radiata (aCR, pCR, and sCR), the FA values of the pre-shunt DTI were decreased by 17%, 22%, and 18%, respectively, compared with the mean FA values of the normal controls. Six months after the shunt operation, the FA values were increased and close to the normal control values (less 1 standard deviations in the aCR, pCR, and sCR). The FA values in these ROIs were then decreased at 11 months postshunt compared with the values 6 months postshunt. Subsequently, the FA values were increased again at 24 months postshunt. Specifically, the FA values in the aCR, pCR, and sCR at 24 months postshunt were even greater than the mean FA values of the normal controls. Compared with the controls, the RD values in the corona radiata ROIs before the shunt were increased significantly (more than 50%). Following the shunt intervention, the RD values decreased with time. At 24 months postshunt, the RD values were still far from the normal control values (over 2 standard deviations).

Compared with the controls, the FA value in the aIC was decreased by 34% pre-shunt. After the shunt, these values were increased but still far from the normal control values (more than 17%). Compared with the controls, the RD value in the aIC was increased pre-shunt and decreased postshunt; however, the RD values in the aIC after the shunt were still far from the normal control values. The FA values in the pIC in all stages were similar to those of the controls. The RD value in the pIC was increased by 46% before the shunt. After the shunt, the RD value decreased gradually with time. At 24 months postshunt, the RD values were close to the normal (less than 2 standard deviations).

CSF flow measurement from PC MRI data showed that the flow was increased and trended to the normal level after shunting. That is, before the shunt, the CSF flow was near zero. However, at 6 months postshunt, the flow was increased to 8.12 cm/s, and nearly 2 years after the shunt, the CSF flow increased to 12.4 cm/s.

## DISCUSSION

In this study, we present the results of a longitudinal examination of DTI characteristics in a child who received a CSF shunt for the treatment of hydrocephalus. DTI was used to evaluate WM integrity as a noninvasive biomarker to quantify in vivo injury and post-treatment recovery in children with hydrocephalus. We made several observations. First, the diffusion indices in the patient before the shunt operation were significantly altered (FA decreased and RD increased) in several important WM regions of the CC, IC, and CR. The DTI demonstrated changes in FA and RD that are consistent with the results of previous studies of hydrocephalus.^16–19^ Second, 2 years following the CSF shunt operation, a follow-up DTI demonstrated a trend toward normalization of the FA and RD values. These results agree with the findings of previous DTI follow-up studies of hydrocephalus.^8,16,20^ The CSF flow measurement from the PC MRI also showed a similar trend toward a normal level of the CSF flow. This result was consistent with our DTI result.

DTI is an MRI-based technique that has been used to study various diseases and neurological disorders. FA is the most commonly reported DTI-derived metric in the literature. The FA values are highly sensitive to the microstructural integrity of WM. Additional measurements of RD can enhance the specificity of detecting microstructural alternations. In the present study, we used both FA and AD to investigate the longitudinal changes in a child with hydrocephalus. A previous study using tract-based summary DTI measures showed a significant increase of RD value and decrease of FA in the gCC in children with hydrocephalus.^17^ Similarly, an ROI-based study in pediatric patients with hydrocephalus has found that patients had lower FA and higher RD in the gCC and sCC than controls.^18^ Lower FA values are been consistently found in the corpus callosum in patients with hydrocephalus.^8,16–18^ In the present study, we detected similar findings in the CC before the shunt operation, which implied that myelination in the CC was impaired. This abnormality pattern was also found in the structures that are adjacent to the IC and CR. Previous DTI studies have found mixed FA changes in some WM structures, such as the internal capsule and corona radiate.^16,21–24^ There is a potential bifurcation in the pattern of diffusion abnormalities in patients with hydrocephalus. The inconsistency between the studies might be caused by variations in the ages of the enrolled patients. In the study performed by Yuan et al,^18^ researchers observed that older children with hydrocephalus had a higher FA in the IC and certain very young patients had a lower FA and a higher RD in the IC than controls. This might explain our results because the patient in the present study is very young. Future studies with more samples are needed to further explore this inconsistency.

Following the operation, all DTI parameters showed a trend toward normalization, yet differences from the values of healthy control subjects remained. Six months after CSF shunt surgery, we performed a follow-up DTI that demonstrated an increase in FA and a decrease of RD values for CC, IC, and CR. These changes are likely due to the reduction in intracranial pressure, which reconstitutes the distorted anatomy and partly restores the patient's cognitive functioning. During neuroimaging, this return was manifested as an FA increase and a RD decrease in the brain. Nearly 1 year after the operation, there was an adjustment of the DTI parameters of the patient in the present study. Although the FA values in all ROIs at this stage were decreased compared with the values at 6 months after the operation, the FA values were still larger than the values before the operation. Therefore, we think that the competition between anatomy distortion and its recovery is still present nearly 1 year after the operation. Two years after the operation, the DTI parameters were closer to the normalization values than at any other timepoint. Therefore, the competition between the anatomy distortion and its recovery becomes more balanced over time. The development process also played an important role in the return of health to this patient. These results coincide with the findings of the previous DTI follow-up study of patients with hydrocephalus.^8,16,21,24,25^ CSF flow measurements from PC MRI data showed that the flow was increased and trended to a normal level after shunting. This result was consistent with the DTI results and further confirmed the positive shunting effect on the development of the child. The longitudinal comparison results can also be explained by network theory. The human brain can be considered to be organized as a series of anatomically segregated regions, with all the regions connected by WM fibers that transfer information among them.^26^ Hydrocephalus often presents with regionally specific damage. This damage is also expected to extend to wide areas throughout the brain. A study with graph theory analysis has found that both hydrocephalus groups under investigation, one pre-operatively and the other postoperatively, showed significantly decreased small-worldness compared with that of normal controls.^2^ The normalized clustering coefficients in both patient groups were decreased. Abnormalities of brain network connectivity were detected in children with hydrocephalus at both pre- and post-operative stages. The integration aspect of the network was affected by the shunt operation. The tract integrity was enhanced in the periventricular regions (including the CC, IC, and CR) of the patient after the operation. We can observe that the CSF shunt operation has long-term effects on the return of children's brain function returning. Shunting can grossly reconstitute the distorted anatomy and improve cognitive functioning.

Through the results, we can see that the longitudinal changes of FA in the CR are specific. Two years after the CSF shunt operation, the FA values in the CR of this patient have risen above the mean FA values of the normal controls. It is not clear why the FA in the CR can elevate to such a high level. Further work with a larger sample size will be required to determine whether this change is a specific phenomenon of this case or a common phenomenon in hydrocephalus.

## CONCLUSION

In this case report, we demonstrated that DTI is a useful tool for evaluating longitudinal changes of hydrocephalus following a CSF shunt operation. The DTI indices indicated a dynamic change with time after the shunting. DTI at long-term follow-up after successful CSF shunting demonstrated a trend toward normalization of the FA and RD values.
